# Sonographic Grading of Non-alcoholic Fatty Liver Disease and Its Association With Serum Biomarkers, Echocardiography, and Electrocardiogram

**DOI:** 10.7759/cureus.112256

**Published:** 2026-07-08

**Authors:** Channabasava Reddy, Madan Mohan Babu L, Suresh A, Shiva Prasad, Chirag M Reddy

**Affiliations:** 1 Radiodiagnosis, Vydehi Institute of Medical Sciences and Research Centre, Bangalore, IND

**Keywords:** cardiovascular risk, echocardiography, electrocardiography, non-alcoholic fatty liver disease, ultrasonography

## Abstract

Introduction: Non-alcoholic fatty liver disease (NAFLD) is the hepatic manifestation of metabolic syndrome and is increasingly recognized as a systemic disorder associated with cardiovascular morbidity. Sonographic grading of NAFLD may reflect progressive metabolic and cardiovascular involvement; however, evidence evaluating the association between ultrasonographic severity and serum biomarkers, echocardiographic parameters, and electrocardiographic findings remains limited. This study aimed to evaluate the association between sonographic grading of NAFLD and serum biomarkers, echocardiographic parameters, and electrocardiographic findings.

Materials and methods: This prospective observational study included 96 adults with sonographically diagnosed NAFLD attending a tertiary care center between March 2024 and October 2025. Participants were categorized into Grade 1, Grade 2, and Grade 3 NAFLD based on ultrasonographic findings. Serum lipid profile and liver enzymes were evaluated, followed by transthoracic echocardiography and 12-lead electrocardiography. Continuous variables were compared using one-way analysis of variance with Tukey's post-hoc test, while categorical variables were analyzed using the chi-squared test or Fisher's exact test, as appropriate. A p-value of <0.05 was considered statistically significant.

Results: Increasing sonographic grade of NAFLD was associated with progressive elevations in total cholesterol, triglyceride, low-density lipoprotein (LDL), very-low-density lipoprotein (VLDL), aspartate aminotransferase (AST), and alanine aminotransferase (ALT) levels, together with a significant reduction in high-density lipoprotein (HDL) levels (all p<0.001). Echocardiographic evaluation demonstrated significant increases in interventricular septal thickness, left ventricular mass, left ventricular mass index, left ventricular dimensions, and progressive reductions in ejection fraction and early-to-late ventricular filling velocity ratio (E/A ratio) (all p<0.001). The prevalence of left ventricular diastolic dysfunction increased from 10 (22.7%) in Grade 1 to 18 (52.9%) in Grade 2 and 14 (77.8%) in Grade 3 (p<0.001). QTc prolongation similarly increased from 6 (13.6%) to 11 (32.4%) and 12 (66.7%) across Grades 1-3 (p<0.001). Although P-wave and T-wave abnormalities were more frequent in advanced disease, these associations were not statistically significant.

Conclusions: Increasing sonographic severity of NAFLD is associated with worsening biochemical abnormalities, adverse cardiac remodelling, left ventricular diastolic dysfunction, and QTc prolongation. Ultrasonographic grading, combined with routine biochemical, echocardiographic, and electrocardiographic evaluation, may facilitate the early cardiovascular risk stratification and comprehensive management of patients with NAFLD.

## Introduction

Non-alcoholic fatty liver disease (NAFLD) is one of the most common chronic liver disorders worldwide and is characterized by the excessive accumulation of triglycerides within hepatocytes in the absence of significant alcohol consumption or other secondary causes of hepatic steatosis [1,2]. It is widely regarded as the hepatic manifestation of metabolic syndrome and is strongly associated with obesity, insulin resistance, type 2 diabetes mellitus, dyslipidemia, and hypertension [3]. The clinical spectrum of NAFLD ranges from simple steatosis to non-alcoholic steatohepatitis (NASH), which may subsequently progress to advanced fibrosis, cirrhosis, and hepatocellular carcinoma [4]. As the early stages of the disease are frequently asymptomatic, timely diagnosis is essential to prevent disease progression and associated complications [5].

Abdominal ultrasonography is the preferred first-line imaging modality for the diagnosis of NAFLD because it is non-invasive, widely available, cost-effective, and free from ionizing radiation [6,7]. Sonographic grading of hepatic steatosis is based on hepatic echogenicity, posterior beam attenuation, and visualization of the intrahepatic vessels and diaphragm, allowing classification into mild, moderate, and severe disease [7]. Although ultrasonography has limited sensitivity for detecting mild steatosis, it remains the most practical imaging technique for routine clinical evaluation [8]. In addition to imaging, serum biochemical markers, including liver enzymes and lipid profile, provide valuable information regarding hepatocellular injury and metabolic derangements associated with NAFLD, and their combined assessment improves disease characterization and risk stratification [9].

Beyond hepatic involvement, NAFLD is increasingly recognized as a multisystem disease with significant cardiovascular implications. Cardiovascular disease is the leading cause of mortality among patients with NAFLD, frequently exceeding liver-related mortality [10]. Hepatic steatosis has been associated with structural and functional cardiac abnormalities, including left ventricular (LV) hypertrophy, diastolic dysfunction, impaired systolic function, and alterations in ventricular geometry [11]. Echocardiography provides a comprehensive evaluation of these structural and functional cardiac changes, while electrocardiography facilitates the detection of electrical abnormalities such as QTc prolongation, conduction disturbances, and repolarization abnormalities, which may predispose patients to cardiac arrhythmias and adverse cardiovascular events [12,13]. Early identification of these subclinical cardiovascular abnormalities may facilitate timely intervention and improve long-term outcomes.

Although previous studies have independently evaluated hepatic, biochemical, and cardiovascular abnormalities in patients with NAFLD, limited data have comprehensively evaluated the association between sonographic grades of hepatic steatosis and serum biomarkers, echocardiographic findings, and electrocardiographic abnormalities within the same population [13,14]. Understanding these associations may improve cardiovascular risk assessment and provide a more comprehensive understanding of the systemic manifestations of NAFLD. We hypothesized that increasing sonographic severity of NAFLD would be associated with progressively worsening serum biochemical abnormalities, echocardiographic alterations, and electrocardiographic abnormalities. Therefore, the primary objective of the present study was to evaluate the association between sonographic grades of NAFLD and serum biochemical markers, echocardiographic parameters, and electrocardiographic abnormalities in adults with NAFLD.

## Materials and methods

This prospective observational study was conducted in the Department of Radiodiagnosis at Vydehi Institute of Medical Sciences and Research Centre in Bangalore, Karnataka, India, over a period of 20 months from March 2024 to October 2025. Approval was obtained from the Vydehi Institutional Ethics Committee (VIEC) (approval number: VIEC/2023/APP/PG/23) prior to the commencement of the study, and written informed consent was obtained from all participants before enrolment.

The sample size was calculated using the standard formula for estimating a single population proportion, $\mathrm{n}=\mathrm{Z}^{2}\times\mathrm{p}\times\mathrm{q}/\mathrm{d}^{2}$, where Z=1.96 corresponding to a 95% CI, p=0.16 based on the 16% prevalence of LV diastolic dysfunction among patients with NAFLD reported by Elsawaby et al. [13], q=0.84, and d=7.3% allowable error [13]. The minimum required sample size was 96 participants.

Consecutive adult patients aged 18-70 years who were referred for abdominal ultrasonography or underwent a master health checkup and were diagnosed with NAFLD on abdominal ultrasound were included in the study until the required sample size was achieved. Consecutive sampling was adopted to minimize selection bias. Patients with a history of significant alcohol consumption, cirrhosis, previous liver interventions (including liver biopsy, pigtail insertion, liver transplantation, or hepatectomy), and chronic or acute liver diseases such as autoimmune hepatitis, Wilson's disease, alpha-1 antitrypsin deficiency, celiac disease, hepatobiliary infections, biliary tract disorders, or hepatic malignancies were excluded.

Following enrolment, all participants underwent detailed clinical evaluation and abdominal ultrasonography. Sonographic examination was performed using Samsung V6 and Samsung V8 ultrasound systems (Samsung Medison, Seoul, South Korea) equipped with 5-12 MHz transducers. The diagnosis and grading of NAFLD were confirmed by a senior consultant radiologist. Hepatic steatosis was graded according to standard sonographic criteria based on liver echogenicity, posterior beam attenuation, and visualization of intrahepatic vessels and the diaphragm [7]. All ultrasonographic examinations were performed according to a standardized departmental protocol using predefined sonographic grading criteria. In equivocal cases, image interpretation was reviewed by the senior consultant radiologist, whose assessment was considered final to ensure consistency in grading. Formal assessment of inter-observer or intra-observer agreement was not performed. Participants were subsequently categorized into three groups according to sonographic severity: Grade 1 (mild steatosis), Grade 2 (moderate steatosis), and Grade 3 (severe steatosis).

Venous blood samples were collected as part of the routine master health checkup for the estimation of liver function tests and lipid profile, including aspartate aminotransferase (AST), alanine aminotransferase (ALT), total cholesterol, triglycerides, high-density lipoprotein (HDL), low-density lipoprotein (LDL), very-low-density lipoprotein (VLDL), and the AST/ALT ratio.

All participants subsequently underwent comprehensive transthoracic echocardiography using a Siemens echocardiography system (Siemens Healthineers, Erlangen, Germany). The examination was performed by a certified cardiologist or a senior postgraduate resident in cardiology under direct supervision in accordance with the recommendations of the American Society of Echocardiography [15]. Echocardiographic parameters assessed included interventricular septal thickness in diastole (IVSd), LV mass, LV mass index (LVMI), left ventricular end-diastolic diameter (LVEDD), left ventricular end-systolic diameter (LVESD), left ventricular ejection fraction (LVEF), and transmitral early-to-late ventricular filling velocity (E/A) ratio for the assessment of diastolic function.

A standard resting 12-lead electrocardiogram was also recorded for every participant at a paper speed of 25 mm/s and calibration of 10 mm/mV. Electrocardiographic recordings were obtained by trained technicians and independently interpreted by a cardiologist. QT intervals were corrected using the Bazett formula ($\mathrm{QTc}=\mathrm{QT}/\mathrm{&radic;RR}$). QTc prolongation was defined as >450 ms in men and >470 ms in women. P-wave abnormality was defined as prolonged duration (>120 ms) and/or abnormal morphology suggestive of atrial conduction abnormality, while T-wave abnormality was defined as inversion, flattening, or nonspecific ST-T changes in two or more contiguous leads. Electrocardiographic evaluation included the assessment of corrected QT (QTc) interval, P-wave abnormalities, and T-wave abnormalities. The cardiologists interpreting both echocardiographic and electrocardiographic findings were blinded to the sonographic grade of NAFLD to minimize observer bias.

All data were entered into Microsoft Excel (Microsoft Corporation, Redmond, Washington, United States) and analyzed using IBM SPSS Statistics for Windows, Version 26.0 (IBM Corp., Armonk, New York, United States). Continuous variables were expressed as mean±standard deviation (SD), while categorical variables were summarized as number and percentage (N (%)). One-way analysis of variance (ANOVA) followed by Tukey's post-hoc test was used to compare continuous variables among the three NAFLD grades. Associations between sonographic grades and categorical variables were assessed using the chi-squared test or Fisher's exact test, as appropriate. No participant had missing data for the variables included in the final analysis. A two-tailed p-value of <0.05 was considered statistically significant.

## Results

The study included 96 participants with sonographically diagnosed NAFLD. Most participants were aged 41-70 years (51 (53.1%)), followed by 26-40 years (39 (40.6%)), while only six (6.3%) were aged 18-25 years. Males constituted the majority of the study population (60 (62.5%)). Office workers represented the largest occupational group (24 (25%)), followed by farmers (20 (20.8%)) and individuals engaged in business (17 (17.7%)). Most participants resided in rural areas (58 (60.4%)), had secondary-level education (34 (35.4%)), and were married (79 (82.3%)). Overall, the study population predominantly consisted of middle-aged, married males from rural backgrounds with secondary education (Table 1).

**Table 1:** Baseline demographic characteristics of the study population (N=96) Data are presented as frequency (n) and percentage (%). Baseline demographic variables including age, gender, occupation, residence, educational status, and marital status are summarized descriptively for the study population. N: total number of participants

Variable	Category	N (%)
Age (years)	18-25	6 (6.3%)
	26-40	39 (40.6%)
	41-70	51 (53.1%)
Gender	Male	60 (62.5%)
	Female	36 (37.5%)
Occupation	Farmer	20 (20.8%)
	Laborer	13 (13.5%)
	Driver	10 (10.4%)
	Business	17 (17.7%)
	Office worker	24 (25%)
	Teacher	9 (9.4%)
	Unemployed	3 (3.1%)
Residence	Rural	58 (60.4%)
	Semi-urban	11 (11.5%)
	Urban	27 (28.1%)
Educational status	No formal education	4 (4.2%)
	Primary	25 (26%)
	Secondary	34 (35.4%)
	Graduate	23 (24%)
	Postgraduate	10 (10.4%)
Marital status	Married	79 (82.3%)
	Single	16 (16.7%)
	Widow	1 (1%)

A significant progressive deterioration in biochemical parameters was observed with increasing sonographic grade of NAFLD. The mean total cholesterol, triglyceride, LDL, VLDL, AST, and ALT levels increased significantly from Grade 1 to Grade 3 (all p<0.001), whereas the mean HDL levels showed a significant decline across the three grades (p<0.001). The AST/ALT ratio did not differ significantly among the groups (p=0.145), indicating that changes in transaminase levels occurred without significant alteration in their ratio across disease severity (Table 2).

**Table 2:** Comparison of biochemical parameters across the sonographic grades of NAFLD Data are presented as mean±SD. Comparisons among the three sonographic grades of NAFLD were performed using one-way ANOVA. F values and corresponding p-values are reported. A p-value of <0.05 was considered statistically significant. HDL: high-density lipoprotein; LDL: low-density lipoprotein; VLDL: very-low-density lipoprotein; AST: aspartate aminotransferase; ALT: alanine aminotransferase; SD: standard deviation; NAFLD: non-alcoholic fatty liver disease; ANOVA: analysis of variance

Parameter	Grade 1 (n=44)	Grade 2 (n=34)	Grade 3 (n=18)	F value	P-value
Total cholesterol (mg/dL)	191.14±18.54	209.38±19.63	235.72±21.84	18.87	<0.001
Triglycerides (mg/dL)	155.61±26.47	192.98±29.84	238.67±35.29	37.18	<0.001
HDL (mg/dL)	45.07±5.11	40.70±4.89	39.29±4.26	10.01	<0.001
LDL (mg/dL)	119.13±16.92	133.15±18.11	161.32±21.73	20.99	<0.001
VLDL (mg/dL)	31.13±5.28	38.60±5.96	47.73±7.06	37.05	<0.001
AST (U/L)	34.53±7.81	52.94±10.42	57.77±11.06	67.65	<0.001
ALT (U/L)	42.21±8.42	54.45±9.61	65.93±10.73	36.09	<0.001
AST/ALT ratio	0.88±0.18	1.00±0.21	0.90±0.17	1.97	0.145

Tukey's post-hoc analysis demonstrated significant pairwise differences between Grade 1 and both Grade 2 and Grade 3 for total cholesterol, triglycerides, LDL, VLDL, AST, and ALT (all p<0.05). Comparisons between Grade 2 and Grade 3 remained significant for total cholesterol, triglycerides, LDL, VLDL, and ALT, whereas AST showed no significant difference between these two grades (p=0.134). HDL differed significantly between Grade 1 and the higher grades but not between Grade 2 and Grade 3. Similarly, no significant pairwise differences were observed for the AST/ALT ratio (Table 3).

**Table 3:** Tukey's post-hoc analysis of biochemical parameters Data are presented as pairwise p-values obtained from Tukey's honestly significant difference post-hoc test following one-way ANOVA. Pairwise comparisons were performed between sonographic grades (Grade 1 vs. Grade 2, Grade 1 vs. Grade 3, and Grade 2 vs. Grade 3). A p-value of <0.05 was considered statistically significant. HDL: high-density lipoprotein; LDL: low-density lipoprotein; VLDL: very-low-density lipoprotein; AST: aspartate aminotransferase; ALT: alanine aminotransferase; ANOVA: analysis of variance

Parameter	Grade 1 vs. Grade 2	Grade 1 vs. Grade 3	Grade 2 vs. Grade 3
Total cholesterol	<0.001	<0.001	0.004
Triglycerides	<0.001	<0.001	0.001
HDL	0.001	<0.001	0.289
LDL	0.003	<0.001	0.008
VLDL	<0.001	<0.001	0.002
AST	<0.001	<0.001	0.134
ALT	<0.001	<0.001	0.001
AST/ALT ratio	0.104	0.881	0.211

Echocardiographic evaluation revealed progressive structural and functional cardiac alterations with increasing NAFLD grade. The mean IVSd, LV mass, LVMI, LVEDD, and LVESD increased significantly from Grade 1 to Grade 3 (all p<0.001). Conversely, the mean ejection fraction and E/A ratio progressively decreased with increasing disease severity (both p<0.001), indicating worsening systolic and diastolic cardiac function associated with increasing sonographic severity of NAFLD (Table 4).

**Table 4:** Comparison of echocardiographic parameters across the sonographic grades of NAFLD Data are presented as mean±SD. Comparisons among the three sonographic grades of NAFLD were performed using one-way ANOVA. F values and corresponding p-values are reported. A p-value of <0.05 was considered statistically significant. IVSd: interventricular septal thickness in diastole; LV mass: left ventricular mass; LVMI: left ventricular mass index; LVEDD: left ventricular end-diastolic diameter; LVESD: left ventricular end-systolic diameter; E/A ratio: early-to-late ventricular filling velocity ratio; SD: standard deviation; NAFLD: non-alcoholic fatty liver disease; ANOVA: analysis of variance

Parameter	Grade 1 (n=44)	Grade 2 (n=34)	Grade 3 (n=18)	F value	P-value
IVSd (cm)	1.00±0.08	1.10±0.10	1.17±0.11	12.56	<0.001
LV mass (g)	150.30±17.84	183.40±18.91	210.10±21.25	54.95	<0.001
LVMI (g/m²)	91.82±8.42	103.29±10.27	121.44±12.63	49.90	<0.001
LVEDD (mm)	48.97±3.21	52.07±3.48	52.95±3.56	14.99	<0.001
LVESD (mm)	31.23±2.52	32.94±2.81	34.59±3.02	9.77	<0.001
Ejection fraction (%)	62.75±3.18	59.87±3.42	56.64±3.76	19.85	<0.001
E/A ratio	1.11±0.14	0.99±0.13	0.86±0.12	16.79	<0.001

Tukey's post-hoc analysis showed significant pairwise differences between Grade 1 and both Grade 2 and Grade 3 for all echocardiographic parameters (all p<0.05). Comparisons between Grade 2 and Grade 3 also remained statistically significant for IVSd, LV mass, LVMI, LVESD, ejection fraction, and E/A ratio. However, LVEDD did not differ significantly between Grade 2 and Grade 3 (p=0.624), suggesting a plateau in ventricular dilatation despite progressive disease severity (Table 5).

**Table 5:** Tukey's post-hoc analysis of echocardiographic parameters Data are presented as pairwise p-values obtained from Tukey's honestly significant difference post-hoc test following one-way ANOVA. Pairwise comparisons were performed between sonographic grades (Grade 1 vs. Grade 2, Grade 1 vs. Grade 3, and Grade 2 vs. Grade 3). A p-value of <0.05 was considered statistically significant. IVSd: interventricular septal thickness in diastole; LV mass: left ventricular mass; LVMI: left ventricular mass index; LVEDD: left ventricular end-diastolic diameter; LVESD: left ventricular end-systolic diameter; E/A ratio: early-to-late ventricular filling velocity ratio; ANOVA: analysis of variance

Parameter	Grade 1 vs. Grade 2	Grade 1 vs. Grade 3	Grade 2 vs. Grade 3
IVSd	0.002	<0.001	0.028
LV mass	<0.001	<0.001	0.003
LVMI	<0.001	<0.001	0.001
LVEDD	0.004	<0.001	0.624
LVESD	0.019	<0.001	0.031
Ejection fraction	<0.001	<0.001	0.014
E/A ratio	<0.001	<0.001	0.026

The prevalence of LV diastolic dysfunction increased significantly with increasing sonographic grade of NAFLD, from 10 (22.7%) participants in Grade 1 to 18 (52.9%) in Grade 2 and 14 (77.8%) in Grade 3 (p<0.001), indicating a significant association between disease severity and impaired diastolic function (Table 6).

**Table 6:** Left ventricular diastolic dysfunction across the sonographic grades of NAFLD Data are presented as frequency (n) and percentage (%). The association between the sonographic grade of NAFLD and left ventricular diastolic dysfunction was assessed using the χ² test. χ² values and corresponding p-values are reported. A p-value of <0.05 was considered statistically significant. NAFLD: non-alcoholic fatty liver disease; χ²: chi-squared statistic

Variable	Grade 1 (n=44)	Grade 2 (n=34)	Grade 3 (n=18)	χ² value	P-value
Absent	34 (77.3%)	16 (47.1%)	4 (22.2%)	18.42	<0.001
Present	10 (22.7%)	18 (52.9%)	14 (77.8%)		

QTc interval prolongation also showed a significant association with NAFLD severity (χ²=15.67; p<0.001). Prolonged QTc was observed in six (13.6%) participants with Grade 1 disease, 11 (32.4%) with Grade 2 disease, and 12 (66.7%) with Grade 3 disease, indicating a progressive increase in arrhythmogenic risk with advancing sonographic grade of NAFLD (Table 7).

**Table 7:** QTc interval across the sonographic grades of NAFLD Data are presented as frequency (n) and percentage (%). The association between the sonographic grade of NAFLD and QTc interval prolongation was assessed using the χ² test. χ² values and corresponding p-values are reported. A p-value of <0.05 was considered statistically significant. QTc: corrected QT; NAFLD: non-alcoholic fatty liver disease; χ²: chi-squared statistic

Variable	Grade 1 (n=44)	Grade 2 (n=34)	Grade 3 (n=18)	χ² value	P-value
Normal QTc	38 (86.4%)	23 (67.6%)	6 (33.3%)	15.67	<0.001
Prolonged QTc	6 (13.6%)	11 (32.4%)	12 (66.7%)		

Electrocardiographic waveform abnormalities demonstrated an increasing trend with advancing NAFLD severity; however, these associations were not statistically significant. P-wave abnormalities were observed in four (9.1%) participants in Grade 1, 10 (29.4%) in Grade 2, and three (16.7%) in Grade 3. Similarly, T-wave abnormalities were noted in three (6.8%), seven (20.6%), and four (22.2%) participants in Grade 1, Grade 2, and Grade 3, respectively. Because of the small expected cell frequencies, Fisher's exact test was applied, which demonstrated no statistically significant association between sonographic grade and either P-wave abnormalities (p=0.066) or T-wave abnormalities (p=0.138). Although both abnormalities tended to occur more frequently in higher NAFLD grades, the differences did not reach statistical significance (Table 8).

**Table 8:** Electrocardiographic waveform abnormalities across the sonographic grades of NAFLD Data are presented as frequency (n) and percentage (%). Associations between the sonographic grade of NAFLD and electrocardiographic waveform abnormalities were assessed using Fisher's exact test because the assumptions for the chi-squared test were not met owing to small expected cell frequencies. A p-value of <0.05 was considered statistically significant. NAFLD: non-alcoholic fatty liver disease

Variable	Grade 1 (n=44)	Grade 2 (n=34)	Grade 3 (n=18)	P-value
P-wave abnormality	4 (9.1%)	10 (29.4%)	3 (16.7%)	0.066
T-wave abnormality	3 (6.8%)	7 (20.6%)	4 (22.2%)	0.138

## Discussion

The present study demonstrated that increasing sonographic severity of NAFLD was significantly associated with progressive deterioration in metabolic, biochemical, echocardiographic, and electrocardiographic parameters, supporting the concept that NAFLD is a multisystem disorder extending beyond the liver. A predominance of middle-aged and older adults was observed, with 51 (53.1%) participants belonging to the 41-70-year age group. Males constituted 60 (62.5%) of the study population, consistent with the higher prevalence of NAFLD reported among men due to greater visceral adiposity and metabolic risk. Alkassabany et al. similarly reported an increasing prevalence of fatty liver with advancing age while also highlighting the growing burden of pediatric NAFLD [16]. Likewise, the Indian National Association for Study of the Liver (INASL) position statement emphasized the increasing prevalence of metabolic syndrome and NAFLD with advancing age in the Indian population [17].

A significant graded increase in serum total cholesterol, triglyceride, LDL cholesterol, VLDL cholesterol, AST, and ALT levels, together with a progressive decline in HDL cholesterol, was observed across the sonographic grades of NAFLD. These findings indicate worsening dyslipidemia and hepatocellular injury with increasing hepatic steatosis. Petrović et al. similarly demonstrated significantly higher lipid parameters and a greater prevalence of metabolic syndrome among patients with advanced NAFLD [18]. Zafar et al. also reported a significant association between the ultrasonographic grades of fatty liver and serum triglyceride and LDL levels [19], findings that are consistent with the present study. In the present study, post-hoc analysis demonstrated significant differences between Grade 1 and the higher grades for both AST and ALT, whereas AST did not differ significantly between Grade 2 and Grade 3 disease. Ghamar-Chehreh et al. likewise identified elevated liver enzymes as significant indicators of NAFLD severity, although they concluded that metabolic parameters were stronger independent predictors than transaminases alone [20].

Cardiac structural and functional abnormalities showed a significant stepwise progression with increasing NAFLD severity. IVSd, LV mass, LVMI, LVEDD, and LVESD increased significantly, whereas LV ejection fraction and E/A ratio progressively declined, indicating both cardiac remodelling and early ventricular dysfunction. These findings closely parallel those reported by Trovato et al., who demonstrated increased LV mass and impaired myocardial function in patients with NAFLD [21]. Similarly, the meta-analysis by Goliopoulou et al. confirmed a significant association between NAFLD and increased LVMI together with impaired diastolic function, supporting the hypothesis that metabolic inflammation and myocardial remodelling accompany progressive hepatic steatosis [22].

The prevalence of LV diastolic dysfunction increased markedly from 10 (22.7%) participants with Grade 1 disease to 18 (52.9%) with Grade 2 and 14 (77.8%) with Grade 3 disease, demonstrating a strong association between sonographic severity and impaired ventricular relaxation. These observations are in agreement with the findings of Goliopoulou et al., who reported significantly higher odds of diastolic dysfunction among patients with NAFLD (odds ratio: 2.07) [22]. Increasing myocardial lipid accumulation, chronic low-grade inflammation, oxidative stress, and myocardial fibrosis have been proposed as potential mechanisms linking NAFLD with diastolic dysfunction and the development of heart failure with preserved ejection fraction.

Electrocardiographic evaluation revealed a significant increase in QTc interval prolongation with advancing NAFLD severity, rising from six (13.6%) participants in Grade 1 to 11 (32.4%) in Grade 2 and 12 (66.7%) in Grade 3 disease. These findings suggest increasing electrical instability and arrhythmogenic risk in patients with advanced hepatic steatosis. Although studies directly evaluating QTc prolongation across sonographic grades remain limited, Wang et al. demonstrated that increasing ultrasound severity of fatty liver was independently associated with a greater risk of coronary artery disease and adverse cardiovascular outcomes, reinforcing the systemic cardiovascular implications of progressive NAFLD [23]. In contrast, P-wave and T-wave abnormalities demonstrated an increasing trend with disease severity but did not reach statistical significance. This lack of statistical significance may partly reflect the relatively small number of participants exhibiting these electrocardiographic abnormalities, particularly in Grade 3 disease. Similar observations have been reported by Goliopoulou et al., who emphasized structural cardiac remodelling and diastolic dysfunction as the predominant cardiovascular manifestations of NAFLD rather than routine electrocardiographic waveform abnormalities [22].

Collectively, the present findings demonstrate a consistent association between the increasing sonographic grade of NAFLD and progressive metabolic abnormalities, hepatocellular injury, structural cardiac remodelling, diastolic dysfunction, and QTc prolongation. Comparable graded worsening of cardiometabolic risk factors has also been reported by Pereira et al., who observed increasing cardiovascular risk with advancing hepatic steatosis [24]. Taken together, these findings reinforce the concept that sonographic grading of NAFLD provides valuable information not only regarding hepatic involvement but also regarding the extent of associated systemic and cardiovascular dysfunction.

Strengths and limitations

The strengths of the present study include its comprehensive evaluation of NAFLD by integrating sonographic grading with biochemical, echocardiographic, and electrocardiographic assessment in the same cohort. The prospective study design, standardized ultrasonographic grading, blinding of cardiologists interpreting echocardiographic and electrocardiographic findings to the sonographic grade, and use of appropriate statistical analyses, including Tukey's post-hoc testing, further enhanced the methodological rigor of the study.

Nevertheless, several limitations should be acknowledged. The cross-sectional observational design precludes causal inference, and the single-center study with a relatively modest sample size of 96 participants, including fewer patients with Grade 3 disease, may limit generalizability. Important clinical confounders such as body mass index, diabetes mellitus, hypertension, smoking status, medication use, and insulin resistance indices were not systematically evaluated; therefore, multivariable adjustment for these factors could not be performed. Furthermore, ultrasonography cannot quantify hepatic fibrosis or replace histopathological assessment. Transient elastography was not performed because the study was designed to evaluate conventional ultrasonographic grading, which remains the most widely available first-line imaging modality in routine clinical practice; additionally, the limited availability of elastography during the study period precluded its routine use. Formal assessment of inter-observer or intra-observer agreement for ultrasonographic grading was also not performed, which may have introduced some degree of observer variability. Advanced metabolic biomarkers such as insulin resistance indices were not evaluated, and residual confounding related to medications and other metabolic factors cannot be completely excluded. In addition, the relatively small number of participants with certain electrocardiographic abnormalities may have limited the statistical power to detect significant associations for P-wave and T-wave abnormalities. Finally, the absence of multivariable regression analysis limits the ability to determine whether the observed associations were independent of potential confounding variables.

## Conclusions

Increasing sonographic severity of NAFLD was significantly associated with progressive worsening of lipid abnormalities, liver enzyme derangements, structural and functional cardiac changes, and electrocardiographic abnormalities, particularly LV diastolic dysfunction and QTc prolongation. These findings highlight that NAFLD is a systemic metabolic disorder with important cardiovascular implications rather than an isolated hepatic disease. Sonographic grading, combined with routine serum biochemical assessment, echocardiography, and electrocardiography, may aid in the early identification of patients with a higher cardiovascular risk profile and facilitate appropriate clinical risk stratification and multidisciplinary management. However, given the observational cross-sectional design of the present study, these findings should be interpreted as associations rather than evidence of causality. Larger prospective multicenter studies incorporating transient elastography and other advanced imaging modalities, comprehensive assessment of metabolic risk factors, and long-term follow-up are warranted to validate these findings and further evaluate the prognostic significance of cardiovascular involvement associated with different sonographic grades of NAFLD.
